# The Influence of Resin Volume Fraction on Selected Properties of Polymer Concrete

**DOI:** 10.3390/ma17246142

**Published:** 2024-12-16

**Authors:** Jakub Smoleń, Krzysztof Stępień, Mateusz Kozioł, Mateusz Włodarczyk, Tomasz Pawlik, Małgorzata Safuta, Krzysztof Groń, Klaudiusz Fross, Piotr Olesik

**Affiliations:** 1 Faculty of Materials Engineering, Silesian University of Technology, Krasińskiego 8 Street, 40-019 Katowice, Poland; 2 HG Hava, Warszawska 198a, 42-200 Częstochowa, Poland; 3 Department of Structural Engineering, Faculty of Civil Engineering, Silesian University of Technology, Akademicka 5, 44-100 Gliwice, Poland; 4 Faculty of Architecture, Silesian University of Technology, ul. Akademicka 7, 44-100 Gliwice, Poland; 5 Micronization Laboratory, Łukasiewicz Research Network—New Chemical Syntheses Institute, Sowińskiego 11 Street, 44-101 Gliwice, Poland

**Keywords:** polymer concrete, resin, composite, aggregate, concrete, civil engineering, architecture

## Abstract

Polymer concrete is a promising material with applications in construction and architecture; however, guidelines for its design and optimization are not well-established in the literature. This study aimed to evaluate how resin volume fraction and aggregate size distribution affect key properties of polyester polymer concrete, including flexural strength, compressive strength, water absorption, and material cost. Three types of quartz aggregates with different particle size distributions were used, as follows: small (below 0.5 mm, quartz dust), medium (0.2–2.0 mm, quartz sand), and large (2.0–10.0 mm, quartz gravel). The resin volume content varied from 5% to 30%. Differences in apparent density, open porosity, water absorption, flexural strength, compressive strength, and material cost were analyzed as functions of resin volume content and aggregate size. The results showed that apparent density and mechanical properties are positively correlated with resin content for small and medium aggregates; however, in the case of large aggregates, flexural strength decreased when the resin volume content exceeded 20%. A significant reduction in material porosity and water absorption (to ~0.4% and ~0.2%, respectively) was observed at high resin volume fractions.

## 1. Introduction

The future of architecture and civil engineering hinges on the development of new materials. Particularly crucial are materials for sustainable development—those that are ecological and energy-efficient in production. The design of new materials can result from the combination of varied materials that complement each other. Materials with enhanced technical parameters, such as increased thermal insulation properties with reduced thickness are especially desired. New materials for facades and roofs, as well as interior finishing materials, are also expected [1,2].

Polymer concretes, also known as resin concretes, are materials where the cement binder is wholly or partially replaced by a polymer binder, most commonly resin. The most used are epoxy resins, polyester resins, and vinyl ester resins [3]. A common feature of traditional cement concretes and polymer concretes is the curing (bonding) process, accompanied by the transition from a liquid state to a hardened solid. The liquid state allows for shaping products in molds to give the desired shape. Although the curing/bonding mechanisms have a chemical–physical nature, the processes differ for cement concretes and polymer concretes. The bonding of cement concretes is conditioned by a characteristic reaction that occurs after mixing cement with water, known as hydration. During this reaction, hydrated calcium silicates are formed, which densify the structure and, after bonding, form a compact spatial network that provides strength [4]. In resin concretes, the crosslinking reaction of the resin involves polymerization. When a hardener/catalyst is added to the resin, a reaction occurs in which monomers and oligomers link together into long and branched polymer structures, forming extensive spatial networks [5]. The development of knowledge about polymer concretes dates back to the 1950s and 1960s, when research began on alternatives to cement concretes with more advantageous properties and properties suitable for specific applications [6]. Depending on the size and morphology of the aggregate, the resin volume fraction ranges from 10% to 25%. The resin content and its type, the size and type of aggregates, the presence of other components, as well as the manufacturing processes affect the properties of the resulting material [7]. Besides aggregates and resin binder, microfillers are often used in the production of polymer concrete to reduce the number of pores and voids in the material [8,9]. Also, fibrous fillers such as glass fibers [10], carbon fibers [11], polymer fibers [12,13], or natural fibers [14,15] are used, which effectively enhance the mechanical properties.

Polymer concretes play a significant role in contemporary construction. Among the most important advantages of polymer concretes that provide an edge over traditional cement concretes are as follows: a shorter curing time and shorter time to reach maximum mechanical strength, higher mechanical strength, resistance to chemical factors, corrosion resistance, and the ability to produce thin-walled products [16]. Unfortunately, unlike traditional concretes, polymer concretes, due to the presence of the resin binder, have poorer resistance to high temperatures [17] and reduced resistance to photodegradation [18]. Due to a range of advantages, polymer concretes are used for repairing and regenerating architectural elements, producing prefabricated products, pipes, manholes, linear drains, wall claddings, tanks (including those for chemically aggressive liquids), garden ornaments, flooring systems, and even machine parts. Due to the ability to impart a unique appearance and extensive possibilities of shaping specific forms, polymer concretes are gaining popularity in modern design, e.g., in architecture, where they are used as imitations of natural stone such as granite or marble [19].

Problems with selecting the proper materials and component proportions arise depending on the application and expected properties. Finding the optimal composition is often preceded by multiple trials, as improving some properties can negatively affect others. For example, achieving lower-density concretes often leads to increased porosity and water absorption, which can deteriorate the material’s frost resistance under usage conditions [20]. Optimization of concrete composition has been described in numerous scientific papers [21,22,23,24,25,26]. Akalin et al. optimized the dosage of the chemical admixture in cement mortars to obtain maximum compressive strength at minimum cost [21]. Niharika et al. used a mix design strategy in the production of geopolymer concrete. They correlated six variables (such as precursor content, curing temperature and time, and NaOH molarity) to optimize the compressive strength; this approach allowed them to obtain a geopolymer with values over 110 MPa [22]. Muthukumer and Mohan were aiming at optimizing resin mass content and curing time for different furan resins as well as aggregate mix proportions. They obtained the polymer concrete with a minimum void content and high compressive strength [23]. Aliha et al. used the Taguchi design of experiment methods to optimize epoxy-based polymer concrete fracture toughness. With this approach, they were able to estimate the combination of resin, filler, and fiber content with the highest fracture energy [26].

The referred research studies prove that there is no single effective path for optimizing the composition of polymer concrete, and the concept of optimal composition or optimal technology depends on many factors. The large-scale use of polymer concretes as an alternative to traditional cement concretes is mainly limited by the excessive cost due to the higher price of resin binder compared to the easily accessible and cheap cement [3,26]. Increasing awareness of product life cycles leads to situations where the customers are guided not only by the price but also by its durability (longevity in the usage environment) and the set of features it possesses. Increased corrosion resistance, greater resistance to chemical agents, low water absorption, and the absence of maintenance needs contribute to the growing interest in polymer concretes and paradoxically can lead to potential savings in the long-term use of these construction materials.

The production processes and technologies used in manufacturing polymer concretes, although characterized by simplicity and low investment required for equipment, must meet specific conditions and be adapted to resins. The appropriate selection of resins and hardeners allows for significantly extending the processing time and avoiding time constraints. A potential issue in large-scale polymer concrete production processes can be the proper mixing system, ensuring proper resin preparation and mixing with aggregates. Aggregates added to uncured resins must be dry [27], and the production process itself must be carried out at a specific temperature (for resins, this temperature corresponds to room temperature). Polymer concrete is an excellent material to produce, for example, prefabricated elements [28,29] but certain construction application areas may be limited because ensuring proper controlled conditions, especially temperature and humidity, can be difficult.

What is of fundamental importance is the matrix resin content in polymer concrete. It is the increase in the resin content that increases the final cost of the material, but at the same time, improves its mechanical properties and tightness [9,17]. Tightness is particularly important due to the potential of polymer concrete to encapsulate various waste components. Optimizing the resin content in polymer concrete requires a large amount of data and a large amount of experimental verification. Therefore, undertaking such work is very purposeful and desirable [23,25,26].

Therefore, this research work is dedicated to the process of optimizing the composition of polymer concretes for three types of quartz aggregates with varying particle sizes. The conducted research aims to demonstrate how the amount of resin matrix affects the properties of polymer concretes and to determine the optimal fractions of resin and aggregates to achieve specific parameters. This study analyzed the impact of polymer concrete composition on flexural strength, compressive strength, material density, porosity, water absorption, and cost. The correlation of the obtained results allows for the identification and description of optimal parameters, which are a valuable source of knowledge for future researchers and manufacturers of polymer concrete products.

The results presented in this article address the research gap by providing detailed design guidelines for optimizing the composition of polymer concrete (resin concrete) in terms of mechanical strength, water impermeability, and material costs, tailored to specific applications. The optimization focuses on a comprehensive analysis of the resin volume fraction and the size of quartz aggregates typically used in concrete production.

## 2. Materials and Methods

**Figure 1:**
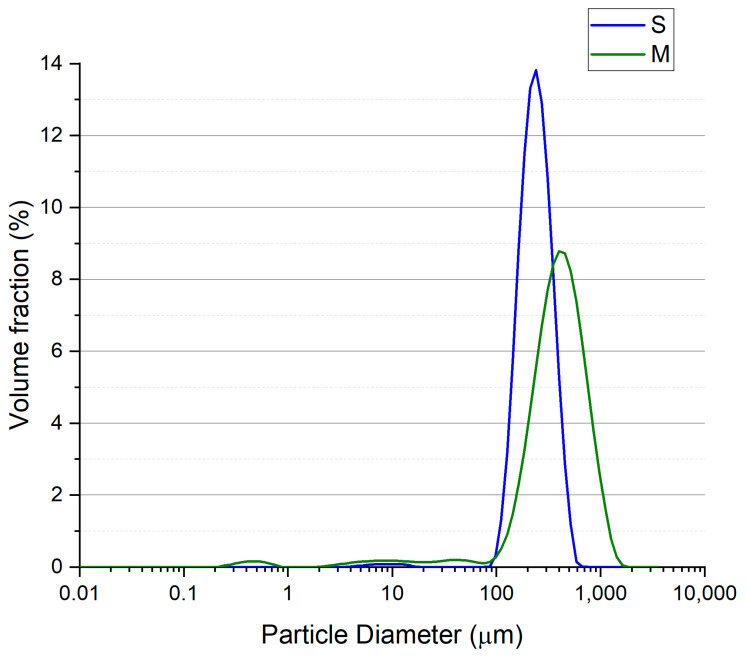
Particle size distribution for S (small)- and M (medium)-series quartz aggregates.

**Figure 2:**
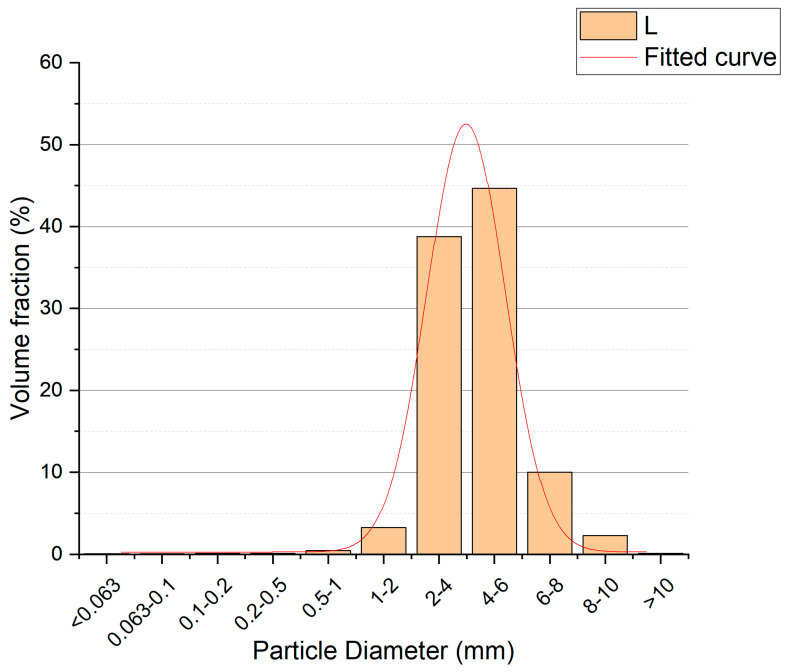
Particle size distribution for L (large)-series quartz aggregates.

**Figure 3:**
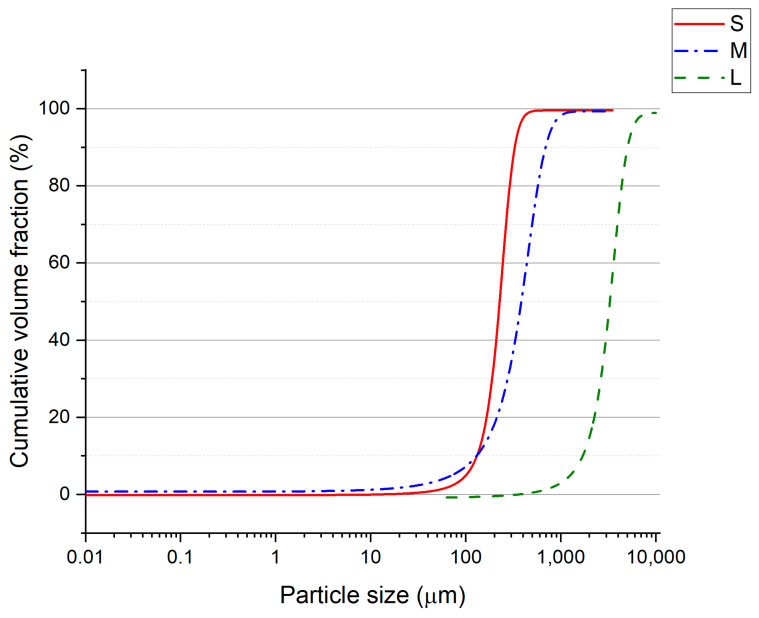
Cumulative curve for quartz aggregates applied within the study: S—small, M—medium, L—large.

Quartz aggregates (QA) with different sizes were acquired from a local market (Kreisel, Poland). Three different fractions of QA were used, as follows: <0.5 mm (S—small size, quartz dust), 0.2–2.0 mm (M—medium size, quartz sand), and 2.0–10.0 mm (L—large size, quartz gravels). The particle size distribution (PSD) analysis of the obtained aggregates was performed. The laser diffraction method on a Malvern Mastersizer 3000 (Malvern Panalytical, Malvern, UK) with Hydro EV wet dispersion unit was used for series S and M, the results are shown in Figure 1. The series L was analyzed with a sieve method using a Multiserw LPzE-2e vibratory sieve shaker (Multiserw-Morek, Marcyporęba, Poland) and the results are presented in Figure 2. The mean diameters of the quartz aggregates were 0.266, 0.481, and 4.200 mm for the S, M, and L series, respectively. Figure 3 shows the cumulative curves of the S-, M-, and L-series quartz aggregates. The packing density of used aggregates was determined. The values were 1.610 ± 0.011, 1.609 ± 0.011, and 1.555 ± 0.013 g/cm^3^ for the S, M, and L series, respectively.

The measurement uncertainty in the test results was calculated using the propagation of uncertainty method, taking into account the known measurement uncertainties of the devices, such as laboratory scales.

Terephthalate polyester resin with increased chemical resistance ESTROMAL 14.PB–06 (LERG, Pustków, Poland) was used as the matrix material. The resin was chosen as the matrix due to its desirable mechanical properties and low environmental impact: the resin is produced from PET-waste materials. The resin was mixed with a cobalt accelerator (ILT Elżbieta Szymczak, Murowana Goślina, Poland) and METOX–50W catalyst (Oxytop Sp. z o. o., Stęszew, Poland). The mass ratio of the resin, accelerator, and catalyst was 100:0.1:1. 

Determination of the maximum matrix resin content was performed. The mold was filled with the respective aggregate and then resin mixture was poured onto it until all the free space was filled (a meniscus appearance). A similar approach to the gravity pouring method was used by Zhou et al. for prepacked aggregate–concrete preparation [30]. The resin and aggregate fill the entire mold, and after adding more, it was poured out. That is why we define it as the “maximum resin content”. After the samples were cured, the maximum resin content was assessed by the mass difference between the concrete and aggregates used to produce it. The calculation results are presented in Table 1. The lowest resin volume was estimated for quartz dust (S) at around 20 vol%, while aggregates M and L were characterized with maximum resin contents at ~25 vol% and ~30 vol%, respectively. This is explained by the differences in the bulk density of the used aggregates. Fine particles have less available space for the resin to infiltrate, and coarser particles have more space. It is worth noting that in polymer concrete, a higher resin content corresponds with better mechanical properties and better tightness.

The previously dried aggregates were mixed with the prepared resin matrix mixture and cast into the rectangular silicone mold (40 × 40 × 160 mm). Cast samples were left at room temperature for 24 h and then post-cured at 60 °C for 24 h in a laboratory drier. The prepared polymer concrete samples varied in terms of resin content and type of QA used. The sample compositions are presented in Table 2. For each series, 3 samples were prepared.

The polymer concrete’s surface morphology was inspected using a Nikon SMZ 745T (Nikon, Tokyo, Japan) stereoscopic microscope. The apparent density, open porosity, and water absorption were characterized with the water displacement method (Archimedes’ method). The flexural strength and compression strength were characterized in accordance with the PN EN 196-1 standard [31]. The mechanical test was performed with Controls^®^ Model 65-L27C12 (Newtown, UK). In the three-point bending test, the support span was 100 mm and load rate was 50 N/s. For the compression test, 40 × 40 mm pads and a load rate of 2400 N/s were used.

For customers and producers, one of the most important criteria is price per unit of material. Resin-based polymer concretes exhibit higher cost per volume than ordinary Portland cement-based concretes. The cost of producing 1 m^3^ of polymer concrete was analyzed using only the prices of substrates used (resin and aggregates). In our calculations, the cost of worker labor, machine operation, etc., was omitted purposely as it highly depends on the local market. To calculate the price, we used the following equation:

(1) Price=aggregates amount·bulk density·price+resin amount density price

(2) $$USD\;per\;m^{3}=m^{3}\cdot kg/m^{3}\cdot USD+m^{3}\cdot kg/m^{3}\cdot USD$$

For the calculation, the average price of the aggregates and polyester resin for Central Europe was used. It was assumed that the purchase price of 1 ton of polyester resin is USD 3000, 1 ton of quartz dust—USD 7, 1 ton of quartz sand—USD 5, 1 ton of quartz gravel—USD 6 [source: producers’ offers available on the Internet and from own inquiries]. Results of the price calculation are presented in Table 3.

## 3. Results and Discussion

### 3.1. Archimedes’ Test Results

The results of apparent density, open porosity, and water absorption and their uncertainties in parentheses are shown in Table 4.

**Figure 4:**
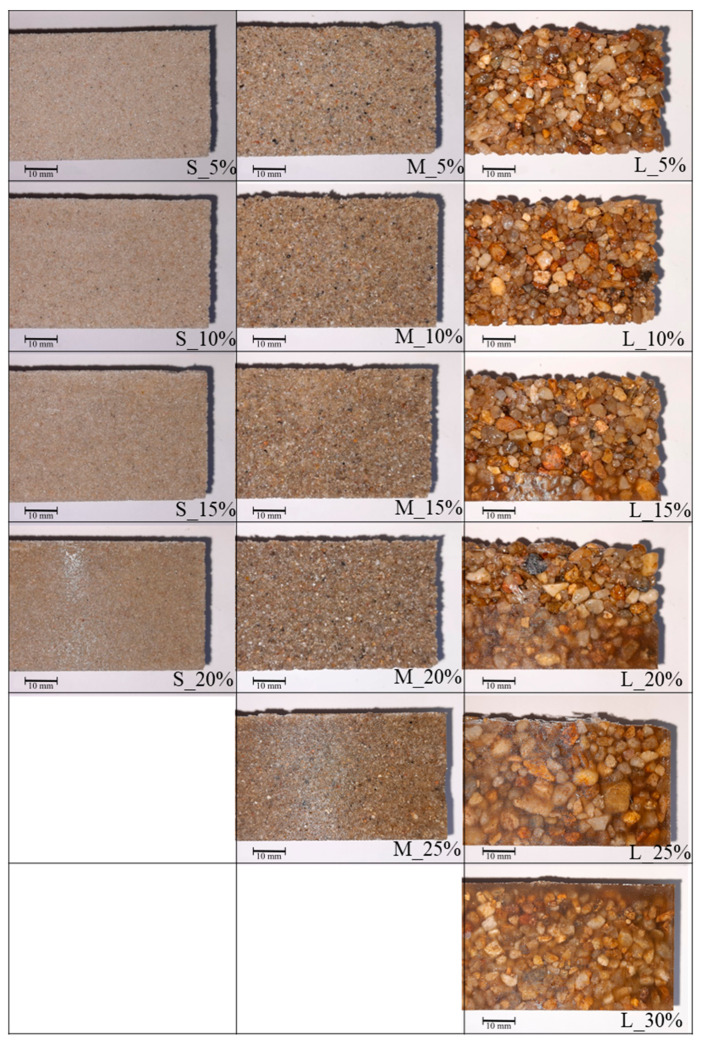
Surface morphology of polymer concretes (Nikon SMZ 745T stereoscopic microscope).

The density of the produced polymer concrete samples was observed within the range of 1.74–2.10 g/cm^3^ with a mean value for all samples of 1.96 g/cm^3^. Polymer concretes S 5–15% and M 5–15% can be classified as lightweight concrete [32], while series L can be classified as regular concrete. The density value is correlated with the resin content and type of QA. For quartz dust and sand (S and M), the density increases with higher resin content. Despite this, the resin density is approximately 2 times lower than quartz (~2.3 g/cm^3^), and the polymer concrete becomes denser at a higher matrix content due to lowering the open porosity. A different effect was observed for quartz gravel as aggregates. For samples with 5–20%, the density does not change, while the open porosity is decreasing; however, when the resin content is above 20%, the density starts to decrease significantly, which suggests it reaches an optimal resin content for sample L_20%. This implies that in polymer concrete with coarse aggregates, the resin easily connects particles with each other, but above a certain matrix content, it starts to overflow and form a pure resin area, which starts to contribute more to the density of the whole material. This was proven by macrostructural analysis, shown in Figure 4. There is a resin layer visible at the concrete bottom in the macrographs for sample L_15%. The layer increases with the increasing resin content and eventually forms a layer on the top. This is the result of gravitational resin flow during production. For series S and M, the impact of the resin volume’s fraction changes is visible. The smallest volume fraction of the resin of 5% allows the production of polymer concrete but its structure shows low compactness, and the use of even a small force leads to material loss, especially at its edges as a result of aggregate chipping. By increasing the content of resin, the material shows better consolidation, reproduction of shape, and clear edges. Also, the surface of the concrete becomes smoother, which results in greater gloss. The above-mentioned observation leads to the conclusion that the concrete structure becomes more compact and sealed with increasing resin volume fraction, regardless of the aggregate type. These results are supported by water absorption changes, which are correlated with resin content. By introducing a sufficient volume fraction of the resin, the concrete ability to absorb water decreased up to ~0.2% for all samples. It is also worth noting that the lower packing density exhibited by coarse aggregates (L series) results in lower open porosity and water absorption with a low resin content; this can be explained by the different structure of this type of polymer concrete.

**Figure 5:**
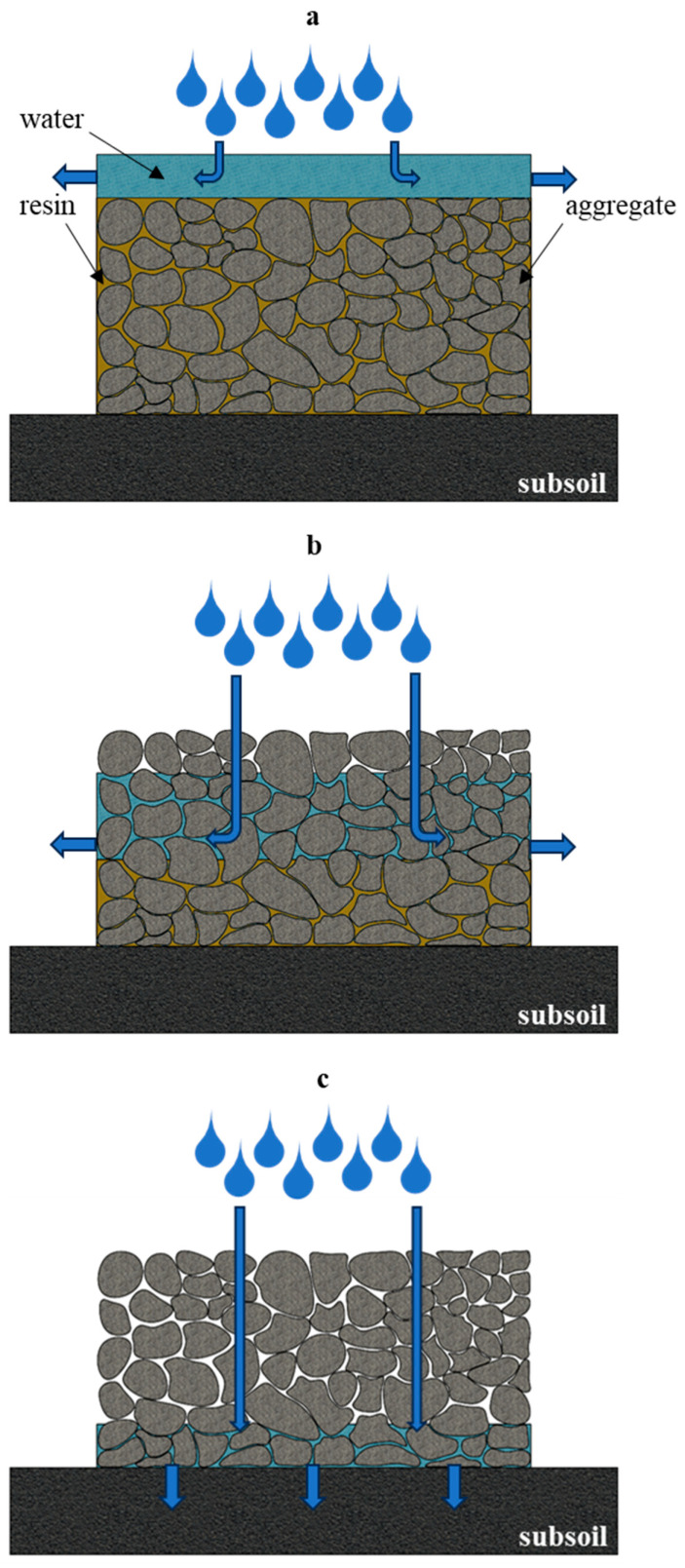
Models of polymer concrete behavior in contact with a liquid: (**a**) fully sealed model (impermeable to liquid); (**b**) a model of partially permeable polymer concrete; (**c**) a model of polymer concrete completely permeable to liquids.

Optimization of the technological parameters (diameter of reinforcement particles and/or matrix volume fraction) of the polymer concrete production process allows for a change in important performance parameters of the product (e.g., open porosity of the product). The type of pores, their size, and the way they are distributed in the volume of the material allow for modeling of functional properties such as, e.g., water permeability. Figure 5 schematically illustrates three models of pore distribution and their influence on the behavior of liquids in contact with the material. The first proposed model (Figure 5a) shows a material that is completely impermeable to liquids due to minimal or even a lack of open porosity. A proposal for the development of such a solution could be materials used in areas where frost resistance is needed, for example, unroofed pavements or materials used for the exterior parts of building walls. The model in Figure 5b shows a polymer concrete with a partially permeable characteristic. The surface layer of the material has a high open porosity, and the lower layer (containing a larger amount of the matrix that flows by gravity to the bottom during curing) is characterized by a tight structure and lack of porosity. This type of solution means that the liquid does not remain on the surface but penetrates deeper; however, it does not penetrate the substrate as the bottom layer is sealed and allows the liquid to drain horizontally. This solution can be used in places whose characteristics do not allow the use of traditional linear drains. The last model proposed (Figure 5c) shows a polymer concrete with a completely permeable character (a large number of open–through pores), where the liquid does not stay on the surface but is transported through the material directly to the substrate. Such a solution can be used in large areas such as parking lots, where it is important to maintain the natural flow of rainwater (absorption and evaporation from a substrate). This kind of solution can have many advantages: the surface is paved, there is no water retention on the surface, and the natural water circulation is not disturbed. A certain limitation of this solution is reduced frost resistance due to increased open porosity.

### 3.2. Mechanical Properties

The results of the mechanical tests are presented in Table 5. The highest mechanical strength (the best obtained values of flexural strength and compressive strength) was observed for samples S_20%, M_25%, and L_30%, for which the flexural strength was 18, 18.3, and 10.3 MPa and the compressive strength was 82.9, 75.6, and 51.7 MPa, respectively. The increase in resin volume fraction in the range of 5–15 vol% corresponds with the slight improvements in mechanical properties; however, after reaching a certain threshold (in the case of series S and M—20%) the sudden improvement in mechanical properties is noticeable. This could be explained with changes in the strengthening mechanism, where resin starts partially transferring the load.

**Figure 6:**
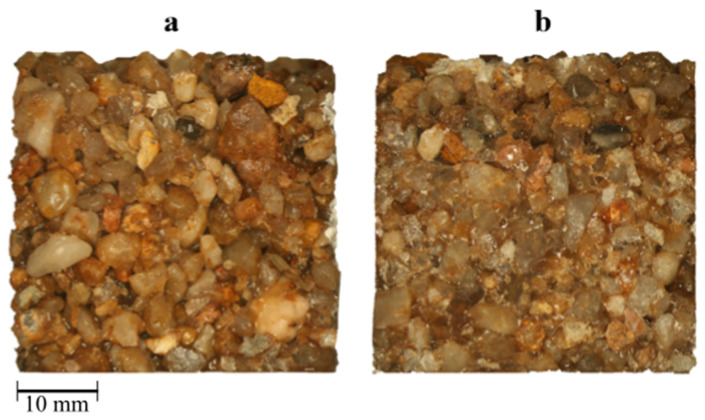
Fracture surface morphology of L-series polymer concrete: (**a**) L_5%, the crack through the grain–matrix boundary; (**b**) L_20%, the crack through the matrix and through the grains.

For large aggregate series L (quartz gravel), a constant increase in mechanical properties is observed with an increase in the volume fraction of the resin, especially in the case of compressive strength. The flexural strength in the range of 15–30% is characterized by the lack of significant changes, which results from the mechanics of material fracture during bending. When the aggregate particles are surrounded by a tight layer of resin (no voids inside the polymer concrete), the external load is transferred to the aggregate particles, which has been observed in material fractures where the crack occurs through the matrix and in some cases even through gravel grains (Figure 6b). In a situation where the resin does not fill the voids between the aggregate particles efficiently, the material breaks along the grain boundary (Figure 6a).

### 3.3. Optimization of the Composition of Polymer Concretes

**Figure 7:**
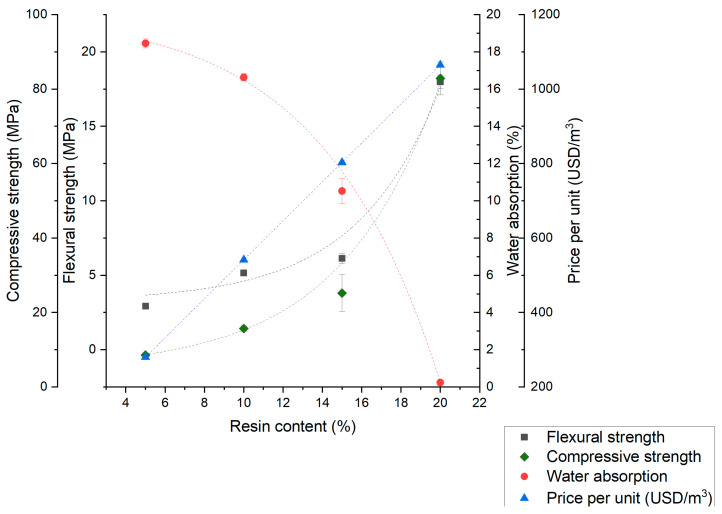
Relationship between the polymer concrete properties and the resin volume fraction for aggregate S (quartz dust).

**Figure 8:**
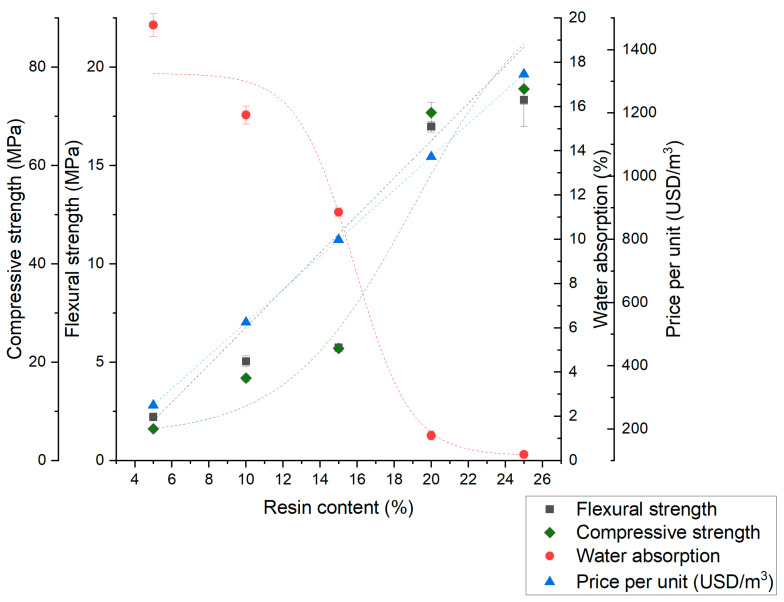
Relationship between the polymer concrete properties and the resin volume fraction for aggregate M (quartz dust).

**Figure 9:**
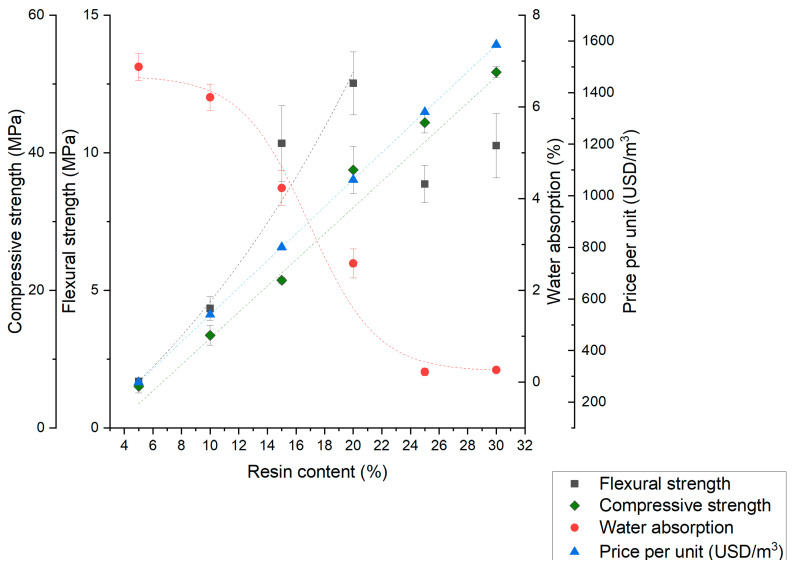
Relationship between the polymer concrete properties and the resin volume fraction for aggregate L (quartz dust).

Based on the experimental results, the overall trends for the dependence of the polymer concrete samples’ properties on the resin volume fraction are presented in Figure 7, Figure 8 and Figure 9. With the performed analysis, optimization of desired properties by changing material composition is possible. There is a noticeable trend of improving mechanical properties and concrete tightness for all aggregate types. Limited water absorption has a positive effect on frost resistance; however, with increasing resin content, the price is also increasing. The trends for water absorption in all cases are non-linear, which possibly correspond to both the void content and polymer concrete’s compactness. In the case of mechanical properties, the non-linearity for the S and M series can be explained with changes in the strengthening mechanism. 

In the L series, the compressive strength has a linear correlation with resin volume fraction as well as flexural strength up to 20%. In the resin volume fractions exceeding 20%, the sudden drop in flexural strength is noticeable, which can be explained with crack propagation in the pure resin layers, displayed and described in Figure 4 and Figure 6. The presented results confirm that the optimization of the materials’ parameters for suitable applications is both possible and promising.

Table 6 compares the research results obtained in this study with those reported by other researchers in the literature. In all studies, a clear trend is observed: an increase in the resin content, regardless of its type (epoxy resin or polyester resin), leads to an improvement in mechanical strength, both in terms of compressive strength and bending/tensile strength. This can be attributed to the resin effectively binding the aggregate particles and acting as a composite matrix that efficiently transfers stress. Maintaining structural continuity, free of voids, enhances cohesion, which is reflected in an increase in density at a constant volume of concrete. Filling the voids between the aggregates enables the even distribution of stress in the polymer concrete. In addition to the resin content, the structural packing of the aggregates is also crucial. Combining coarse and fine aggregate fractions in a single concrete mix minimizes voids as smaller particles from the fine aggregate fraction fill the spaces between larger particles from the coarse aggregate fraction. This combination of coarse and fine aggregates is beneficial, allowing for higher mechanical strength to be achieved with the same resin content, as confirmed by previous research studies [17,24,25,33,34]. Moreover, there is a noticeable trend indicating that an increase in the proportion of fine aggregate relative to coarse aggregate further enhances the strength of the concrete.

Comparison of the research results obtained in this study with similar studies conducted by Seco and others [35] reveals that vibrating the molds after casting the concrete can be beneficial as it increases the mechanical strength by minimizing internal porosity caused by trapped air bubbles within the concrete. Seco, using vibrations on polymer concrete composed of 25% volume of polyester resin and 75% volume of quartz sand, achieved a flexural strength of 25.8 MPa and a compressive strength of 99.3 MPa. In contrast, in this study, where vibration was not applied, the corresponding strength values for the same composition were 18.3 MPa and 75.6 MPa, respectively. Additional factors that may have contributed to the differences in results include the type of polyester resin used and the interfacial bond quality. Research studies utilizing combinations of aggregates of different sizes demonstrate that the maximum resin volume fraction decreases due to a reduction in the void spaces between aggregate particles that can be filled with resin. For instance, in the works of Jafari et al. [25] and Haidar et al. [24], the combination of sand with coarse aggregate allowed for the incorporation of 13–14 wt% resin into the aggregates. In contrast, in this study, for quartz sand (M), the maximum resin content was 19.5 wt%. The morphology of the aggregate is also important for the maximum resin content, especially the open porosity at the surface, where the increase in porosity increases resin absorption, which allows for the introduction of more resin and increased mechanical strength, as shown in the tests [17,33].

## 4. Conclusions

This research, carried out on polymer concretes with three types of quartz aggregates (S—dust, M—sand, and L—gravels), allowed us to formulate the following conclusions:

- Depending on the aggregate particle size, the optimal resin volume fraction required for non-water-absorbing materials is different. The larger the particle size, the more resin is needed to obtain low water absorption. However, lower packing density results in open pores in cases of low resin content. For the aggregates with a diameter below 0.5 mm, the maximum resin volume fraction making technical sense is 20%, while for the aggregates with a diameter below 2.0 mm and 10.0 mm, it is 25% and 30%, respectively;
- The density and mechanical properties are positively correlated with the resin volume fraction, while open porosity and water absorption are negatively correlated with it. This suggests that the maximum values of the mechanical properties are achieved in polymer concretes with a sealed, tighter structure, where resin is not only the binder but also acts as a composite matrix carrying some part of the load;
- Appropriate selection of the volume fraction of the resin allows us to obtain specific properties of polymer concretes, including obtaining materials that are completely impermeable to liquids, partially permeable, or fully permeable, depending on the area of application. Polymer concretes, despite the higher price of input materials in relation to traditional cement concretes, show a number of significantly better properties that can compensate for the higher price. Among other applications, they can serve as an encapsulating medium for dangerous waste.

This article may be of key importance to manufacturers in the production of polymer concrete products and future research on this material. The correlations of the obtained results will help to achieve the desired material parameters in ongoing research and industrial production, which will reduce the time to prepare a suitable polymer concrete composition. The results of the conducted research can also be helpful in the study of encapsulation of hazardous waste in this type of material.

## Figures and Tables

**Table 1:** Calculation results of maximum resin content (the uncertainties are presented in parenthesis).

	Aggregate Mass [g]	Sample Mass [g]	Resin Mass [g]	Resin Density [g/cm^3^]	Resin Volume [cm^3^]	Sample Volume [cm^3^]	Maximum Resin Content [vol%]
Aggregate S	378.7 (0.1)	438.3 (0.1)	59.6 (0.2)	1.17	50.94 (0.17)	256	19.9 (0.07)
Aggregate M	370.6 (0.1)	447.5 (0.1)	76.9 (0.2)	1.17	65.72 (0.17)	256	25.7 (0.07)
Aggregate L	399.2 (0.1)	490.8 (0.1)	91.6 (0.2)	1.17	78.29 (0.17)	256	30.6 (0.07)

**Table 2:** Sample composition and designation.

Sample	Aggregate S (Fraction Below 0.5 mm) [vol%/mass%]	Aggregate M (Fraction 0.2–2.0 mm) [vol%/mass%]	Aggregate L (Fraction 2.0–10.0 mm) [vol%/mass%]	Resin Content [vol%/mass%]
S_5%	95/96.3	-	-	5/3.7
S_10%	90/92.5	-	-	10/7.5
S_15%	85/88.6	-	-	15/11.4
S_20%	80/84.6	-	-	20/15.4
M_5%	-	95/96.3	-	5/3.7
M_10%	-	90/92.5	-	10/7.5
M_15%	-	85/88.6	-	15/11.4
M_20%	-	80/84.6	-	20/15.4
M_25%	-	75/80.5	-	25/19.5
L_5%	-	-	95/96.2	5/3.8
L_10%	-	-	90/92.3	10/7.7
L_15%	-	-	85/88.2	15/11.8
L_20%	-	-	80/84.1	20/15.9
L_25%	-	-	75/79.9	25/20.1
L_30%	-	-	70/75.6	30/24.4

**Table 3:** Component material prices for 1 m^3^ of polymer concrete.

Sample	Price, [USD per m^3^]	Sample	Price, [USD per m^3^]	Sample	Price, [USD per m^3^]
S_5%	280	M_5%	275	L_5%	278
S_10%	542	M_10%	537	L_10%	539
S_15%	803	M_15%	799	L_15%	801
S_20%	1065	M_20%	1061	L_20%	1063
		M_25%	1322	L_25%	1324
				L_30%	1586

**Table 4:** Polymer concrete apparent density, open porosity, and water absorption results.

Sample	Apparent Density, [g/cm^3^]	Open Porosity, [%]	Water Absorption, [%]
S_5%	1.78 (0.01)	32.84 (0.59)	18.45 (0.24)
S_10%	1.81 (0.02)	30.02 (0.25)	16.63 (0.19)
S_15%	1.93 (0.01)	20.33 (1.14)	10.52 (0.67)
S_20%	2.03 (0.02)	0.48 (0.02)	0.24 (0.01)
M_5%	1.74 (0.02)	34.17 (0.57)	19.68 (0.52)
M_10%	1.82 (0.01)	28.36 (0.7)	15.62 (0.41)
M_15%	1.90 (0.01)	21.35 (0.3)	11.23 (0.19)
M_20%	2.06 (0.01)	2.33 (0.41)	1.13 (0.2)
M_25%	2.09 (0.01)	0.57 (0.10)	0.27 (0.05)
L_5%	2.11 (0.01)	14.53 (0.69)	6.87 (0.3)
L_10%	2.09 (0.03)	13.01 (0.74)	6.21 (0.29)
L_15%	2.10 (0.02)	8.91 (0.78)	4.24 (0.38)
L_20%	2.09 (0.02)	5.39 (0.61)	2.59 (0.32)
L_25%	2.03 (0.01)	0.45 (0.17)	0.22 (0.08)
L_30%	1.95 (0.02)	0.52 (0.02)	0.26 (0.01)

**Table 5:** Results of polymer concrete mechanical testing.

Sample	Flexural Strength, [MPa]	dev [MPa]	Compressive Strength, [MPa]	dev [MPa]
S_5%	2.9	0.2	8.6	0.6
S_10%	5.2	0.1	15.7	0.7
S_15%	6.1	0.3	25.2	4.9
S_20%	18.00	0.5	82.9	4.4
M_5%	2.2	0.1	6.4	0.1
M_10%	5.1	0.3	16.8	0.7
M_15%	5.8	0.1	22.8	0.8
M_20%	17	0.3	70.7	2
M_25%	18.3	1.4	75.6	3.2
L_5%	1.7	0	6.0	1
L_10%	4.3	0.4	13.5	1.4
L_15%	10.3	1.4	21.5	0.4
L_20%	12.5	1.2	37.5	3.5
L_25%	8.9	0.7	44.4	1.5
L_30%	10.3	1.2	51.7	0.8

**Table 6:** Comparison of results.

Polymer Concrete	Resin Content	Flexural Strength [MPa]	Compressive Strength [MPa]	Water Absorption [%]	Reference
Epoxy resin, Limestone + river sand (50:50)	10 wt%	6.90	34.9	-	[25]
	12 wt%	7.93	49.1	-	
	14 wt%	8.19	54.0	-	
Epoxy resin, Siliceous sand + gravel (75:25)	5 wt%	~5.0	~36.0	5.67	[24]
	9 wt%	~24.0	~60.0	0.39	
	13 wt%	~27.0	~70.0	0.14	
Polyester resin, Lightweight Expanded Clay Aggregate + river sand (70:30)	15 wt%	~5.0	~20.0	~2.0	[17]
	18 wt%	~6.0	~25.0	~1.5	
	21 wt%	~8.0	~30.0	~0.5	
	24 wt%	~14.0	~55.0	~0.2	
	27 wt%	~14.0	~55.0	~0.2	
Polyester resin, Lightweight Expanded Clay Aggregate + river sand (70:30)	15 wt%	-	18.83	-	[33]
	18 wt%	-	23.92	-	
	21 wt%	-	32.63	-	
	24 wt%	-	52.43	-	
	27 wt%	-	54.04	-	
Polyester resin, Siliceous sand, + gravel	15 wt%	15.98	78.71	-	[34]
	20 wt%	13.86	82.3	-	
	25 wt%	14.95	84.92	-	
Polyester resin, Siliceous sand	20 vol%	22.0	93.4	-	[35]
	25 vol%	25.8	99.3	-	
	30 vol%	25.1	106.2	-	
Polyester resin, Siliceous sand (S)	5 vol%/3.7 wt%	2.9	8.6	18.45	This work
	10 vol%/7.5 wt%	5.2	15.7	16.63	
	15 vol%/11.4 wt%	6.1	25.2	10.52	
	20 vol%/15.4 wt%	18.0	82.9	0.24	
Polyester resin, Siliceous sand (M)	5 vol%/3.7 wt%	2.2	6.4	19.68	This work
	10 vol%/7.5 wt%	5.1	16.8	15.62	
	15 vol%/11.4 wt%	5.8	22.8	11.23	
	20 vol%/15.4 wt%	17	70.7	1.13	
	25 vol%/19.5 wt%	18.3	75.6	0.27	
Polyester resin, gravel (L)	5 vol%/3.7 wt%	1.7	6.0	6.87	This work
	10 vol%/7.5 wt%	4.3	13.5	6.21	
	15 vol%/11.4 wt%	10.3	21.5	4.24	
	20 vol%/15.4 wt%	12.5	37.5	2.59	
	25 vol%/19.5 wt%	8.9	44.4	0.22	
	30 vol%/24.4 wt%	10.3	51.7	0.26	

## Data Availability

The original contributions presented in this study are included in the article, further inquiries can be directed to the corresponding author.
